# Utilization of the Parenteral Morphine in Emergency Department using the Anatomical Therapeutic Chemical Classification/Defined Daily Doses (ATC/DDD) System

**DOI:** 10.30476/BEAT.2020.86225

**Published:** 2020-07

**Authors:** Farzad Bozorgi, Ebrahim Salehifar, Seyed Mohammad Hosseininejad, Siavash Moradi, Ghazaleh Janbazi, Aroona Chabra

**Affiliations:** 1 *Orthopedic Research Center, Mazandaran University of Medical Sciences, Sari, Iran*; 2 *Pharmaceutical Research Center, Mazandaran University of Medical Sciences, Sari, Iran*; 3 *Diabetes Research Center, Mazandaran university of Medical Sciences, Sari, Iran*; 4 *Faculty of Medicine, Mazandaran University of Medical Sciences, Sari, Iran*; 5 *Student Research Committee, Faculty of Medicine, Mazandaran University of Medical Sciences, Sari, Iran*; 6 *Student Research Committee, Faculty of Pharmacy, Mazandaran University of Medical Sciences, Sari, Iran*

**Keywords:** Rational utilization, Morphine, Emergency department (ED), Anatomical Therapeutic Chemical Classification/Defined Daily Doses (ATC/DDD).

## Abstract

**Objective:**

To evaluate the utilization of the parenteral morphine in Emergency Department (ED) using the Anatomical Therapeutic Chemical Classification/Defined Daily Doses (ATC/DDD) system.

**Methods:**

In this retrospective cross-sectional study, morphine administration was recorded in 4-year time period from January 2013 to December 2016 in the ED of a referral center. The dose of the administered morphine was evaluated using the ATC/DDD system. The ATC/DDD of the parenteral morphine was calculated based on the world health organization (WHO). The data was evaluated based on the different diagnosis and conditions using the ATC/DDD protocol.

**Results:**

In this study, 500 patients referred to ED with mean age of 48.29 ± 10.10 years were included. There were 306 (61.2%) men and 194 (38.8%) women among the patients. The lowest and highest DDD of parenteral morphine were 0.1 and 0.43, respectively. The utilization of parenteral morphine was significantly higher in men when compared to women (*p*<0.001). Those with history of tricyclic anti-depressant (TCA) consumption (*p*<0.001) and opium addiction (*p*<0.001) had significantly higher parenteral morphine utilization. Those with pain in the extremities and chest pain had significantly higher parenteral morphine utilization (*p*<0.001).

**Conclusion:**

The utilization of parenteral morphine in the ED of our center was higher than the WHO standard dosage. The morphine utilization was associated with male gender, opium addiction and TCA consumption.

## Introduction

Currently, the irrational prescription and use of drugs has been reported to be endemic in many countries, including developing countries such as Iran [1, 2]. It has been reported that about 48% of the administered drugs in the ED are in injectable forms of drugs [3]. Accordingly, per capita consumption of injectable preparations is about 10, while it is about 3.4 in developing countries and much less in developed countries [3, 4]. Rationale use of drugs especially in emergency department (ED) is required in source-limiting hospitals [4]. Experience has shown that even with the selection of a drug from a valid list, there is still a risk of its ineffectiveness, lack of adequate immunity or irrational administration. In the case of effective treatments, the benefits of this system will be present throughout the system [5]. According to the world health organization (WHO), rational usage of drug is referred to administration and taking the drug in line with the clinical needs of the patient, in the amounts based on its needs, for a sufficient period of time and at the lowest possible cost [6].

Many failures in prescribing and taking prescription drugs need to find corrective remedies that are a good way to do drug use evaluation (DUE) studies. DUE studies are done in three ways: retrospective, contemporary, and futuristic. According to the WHO, DUE studies are to achieve rational use of drugs require the use of appropriate therapeutic drugs that can meet the patient's clinical needs with minimal cost and side effects [7]. Successful implementation of the DUE will ensure the proper and effective use of the medication. One of the goals of implementing DUE programs is to ensure that the quality of drug use is reasonable, to provide patients with health, to anticipate and anticipate adverse side effects, to chart drug use patterns in the community, and to reduce waste costs [8]. Drugs, especially their injectable forms, have great utility in relieving acute pain and control of pulmonary edema in emergency situations. One of these drugs is morphine. Morphine is also used as an anesthetic supplement in addition to the analgesic effect. The usual dose is 10 mg every four hours [9, 10].

Several studies have indicated that opioids are inappropriately administered, resulting in inadequate pain relief. Misuse and excessive use of opioid drugs, including morphine, can cause problems such as side effects, drug abuse, and addiction due to irrational drug use [11]. Studies at the global level are very small and data are scarce accordingly [11, 12]. The only studies available in this area were conducted in the United States, Spain and Finland [13, 14]. Due to the lack of reasonable and adequate reports on the use of opioid analgesia for the relief of acute pain in the EDs, this study aimed to investigate the pattern of morphine consumption and the study of the factors associated with the use of this drug in ED of a large referral center.

## Materials and Methods

*Study population*

This was a retrospective cross-sectional study with the aim of DUE of morphine in ED. The study was conducted in ED of the Imam Khomeini hospital, a tertiary healthcare center affiliated with Mazandaran University of Medical Sciences, Sari, Iran, during a 4-year period from January 2013 to December 2016. We have consecutively included patients who were admitted to the EDs of our center during the study period who received morphine ampules as analgesics. We have excluded those patients with incomplete medical records and those with analgesic administration without appropriate indication. The study protocol is approved by the institutional review board (IRB) and medical ethics committee of Mazandaran University of Medical Sciences. As this was a retrospective study being conducted on the medical records, the need for informed written consents was waived by the IRB. The information remained anonymous. 

*Study protocol*

The medical records of the patients were reviewed and the data were recorded in the data gathering forms. The recorded data included age, gender, duration of hospital stay, and reasons for admission that was extracted from the hospital’s health information system (HIS). We also recorded the history of allergy, pre-admission drugs, history of smoking, drugs and alcohol, the cause of admission, the amount of injectable morphine, comparing the order of the physician and the nursing report, and the way of prescribing the nurse and doctor were available in the files. Due to the sensitivity of the nurses and their presence on the patient's bedside, it has been documented in the records.

*ATC/DDD system*

To complete the questionnaire, 500 cases of morphine ampoule recipients in the ED were evaluated. Using the ATC/DDD system, the dose was converted to daily dosage. Calculation of the amount of parenteral morphine consumed based on the ATC/DDD system and DDD/per day unit is based on the following formula:

DDD100>BedDay=Consumed quantity per year(mg)/DDD 365×Number of Beds×Occupancy Index×100

Using the WHO site, the DDD level of morphine drug was extracted and a questionnaire was developed for assessing the morphine ampoule administration process. (Questionnaire Attached)

*Sample size calculation*

According to Vatanpour *et al*., [15] regarding the average consumption of morphine in the emergency department, the information is as follows:

σ=8.5

d=0.73

α=0.05

β=0.2

$n=\frac{{\left(\right)}^{2\sigma^{2}}}{d^{2}}=\frac{7.89\times 5.8}{0.73}\approx 500$

The total sample size was 500 people. For completing the questionnaire form, from each of the four years from January 2013 to December 2016, the case of 125 patients receiving parenteral morphine in the ED was investigated.

*Statistical analysis*

After completing the questionnaires, information on how the drug was consumed in patients with a specific daily intake of morphine reported by the WHO was compared. Data are presented as mean ± SD or proportions as appropriate. All the statistical analysis was conducted using statistical package for social sciences (SPSS Inc., Chicago, Illinois, USA) version 21.0. We compared the proportions using chi-square test. The continuous variables with normal distribution were compared using independent t-test and those without were compared using Mann-Whitney U-test. Chi-square test was used to compare proportions between 4 different years. For parametric variable with and without normal distribution, Pearson and Spearman’s correlations tests were used to assess the linear correlation, respectively. A two-sided p-value of less than 0.05 was considered statistically significant. 

## Results

Overall, we have included 500 patients who referred to our center during the study period. The mean age of the patients was 48.29 ± 10.37 (ranging from 18 to 79) years. Among the patients, there were 306 (61.2%) men and 194 (38.8%) women. Trauma was the most common cause of the admission followed by dyspepsia and flank pain. Table 1 summarized the baseline characteristics of the patients enrolled in the current study.

**Table 1:** The baseline characteristics of the 500 patients admitted to the emergency department

	**2013 (n=125)**	**2013 (n=125)**	**2013 (n=125)**	**2013 (n=125)**	**Total**
**Gender **					
Men (%)	73 (58.4%)	78 (62.4%)	80 (64.0%)	75 (60.0%)	306 (61.2%)
Women (%)	52 (41.6%)	47 (37.6%)	45 (36.0%)	50 (40.0%)	194 (38.8%)
**Age **					
10-19 (%)	4 (3.2%)	6 (4.8%)	5 (4.0%)	4 (3.2%)	19 (3.8%)
20-29 (%)	23 (18.4%)	23 (18.4%)	26 (20.8%)	23 (18.4%)	95 (19.0%)
30-39 (%)	29 (23.2%)	33 (26.4%)	30 (24.0%)	36 (28.8%)	128 (25.6%)
40-49 (%)	19 (15.2%)	23 (18.4%)	18 (14.4%)	20 (16.0%)	80 (16.0%)
50-59 (%)	24 (19.2%)	20 (16.0%)	24 (19.2%)	18 (14.4%)	86 (17.2%)
60-69 (%)	15 (12.0%)	11 (8.8%)	15 (12.0%)	13 (10.4%)	54 (10.8%)
70-79 (%)	8 (6.4%)	5 (4.0%)	5 (4.0%)	7 (5.6%)	25 (5.0%)
≥80 (%)	3 (2.4%)	4 (3.2%)	2 (1.6%)	4 (3.2%)	13 (2.6%)
**Cause of admission**					
Trauma (%)	26 (20.8%)	9 (23.2%)	33 (26.4%)	30 (24.0%)	118 (23.6%)
Dyspepsia (%)	43 (34.4%)	41 (32.8%)	35 (28.0%)	36 (28.8%)	155 (31.0%)
Flank pain (%)	40 (32.0%)	31 (24.8%)	37 (29.6%)	36 (28.8%)	144 (28.8%)
Headache (%)	3 (2.4%)	4 (3.2%)	3 (2.4%)	4 (3.2%)	14 (2.8%)
Chest pain (%)	3 (2.4%)	4 (3.2%)	3 (2.4%)	2 (1.6%)	12 (2.4%)
Back pain (%)	2 (1.6%)	5 (4.0%)	4 (3.2%)	4 (3.2%)	15 (3.0%)
Limb pain (%)	2 (1.6%)	5 (4.0%)	4 (3.2%)	3 (2.4%)	14 (2.8%)
Lethargy (%)	3 (2.4%)	3 (2.4%)	4 (3.2%)	1 (0.8%)	11 (2.2%)
Testis pain (%)	2 (1.6%)	1 (0.8%)	1 (0.8%)	6 (4.8%)	10 (2.0%)
Others (%)	1 (0.8%)	2 (1.6%)	1 (0.8%)	3 (2.4%)	7 (1.4%)

Among the patients, there were 88 (17.6%) smokers, and 25 (5.0%) opium addicts. The rate of alcohol consumption was 15 (3%) in the series. In the toxicology results, 11 (2.2%) patients had positive reports. Overall, 16 (3.2%) and 9 (1.8%) patients had consumed benzodiazepine and tricyclic antidepressants (TCA), respectively. The frequency of drug and substance abuse was comparable between the 4 years of the study. Table 2 reviews the drug and substance abuse frequency in our series. As demonstrated in Table 3, the lowest and highest DDD of parenteral morphine were 0.1 and 0.43, respectively. As the patients in ED were admitted only for 1 day, the DDD/100 bed days with a DDD of 0.14 in the study period was 93.92 ± 22.32. The highest DDD was calculated to be in 2016 with mean of 14.03 ± 38.48. The rates are summarized in Table 3.

**Table 2:** The frequency of drug and substance abuse in a series of 500 patients referring to our emergency department during the study period

	**2013 (n=125)**	**2013 (n=125)**	**2013 (n=125)**	**2013 (n=125)**	**Total**	**p-value** ^α^
Opium (%)	4 (3.2%)	6 (4.8%)	6 (4.8%)	9 (7.2%)	25 (5.0%)	0.426
Methadone (%)	4 (3.2%)	3 (2.4%)	2 (1.6%)	4 (3.2%)	13 (2.6%)	0.516
Smoking (%)	18 (14.4%)	25 (20.0%)	20 (16.0%)	25 (20.0%)	88 (17.6%)	0.553
Alcohol (%)	3 (2.4%)	4 (3.2%)	4 (3.2%)	4 (3.2%)	15 (3.0%)	0.977
Allergy (%)	2(1.6%)	3 (2.4%)	4 (3.2%)	2 (1.6%)	11 (2.2%)	0.796
Benzodiazepine (%)	4 (3.2%)	5 (4.0%)	2 (1.6%)	5 (4.0%)	16 (3.2%)	0.671
TCA^b^ (%)	3 (2.4%)	2 (1.6%)	1 (0.8%)	3 (2.4%)	9 (1.8%)	0.742

^α^Chi-square test; ^b^**TCA:** Tricyclic anti-depressant

**Table 3:** The utilization of parenteral morphine in 500 patients admitted to the emergency department during the study period

			**Morphine Utilization (DDD** ^a^ **)**		**DDD/100 bed days**
**Year**	**Lowest**	**Highest**	**Mean ± SD**	**Medium (Range 25-75%)**	**Mean ± SD**
2013	0.10	0.40	0.14 ± 0.06	0.10 (0.10-0.17)	16.74 ± 37.90
2014	0.10	0.43	0.13 ± 0.06	0.10 (0.10-0.17)	14.84 ± 35.55
2015	0.10	0.33	0.14 ± 0.05	0.10 (0.10-0.17)	13.97 ± 37.40
2016	0.10	0.40	0.14 ± 0.05	0.17 (0.10-0.17)	14.03 ± 38.04
Total	0.10	0.43	0.14 ± 0.06	0.10 (0.10-0.17)	14.93 ± 37.22

^a^ **DDD:** Defined Daily Doses

We have also assessed the utilization of parenteral morphine in different age groups and genders. There was no statistically significant difference between the different age groups regarding the morphine utilization according to DDD in those admitted to ED. However, the utilization of parenteral morphine was significantly higher in men when compared to women (*p*<0.001). The parenteral morphine utilization according to the DDD in different gender and age groups is demonstrated in Table 4. We have also compared the parenteral morphine utilization between different drug and substance abuse categories. As demonstrated in Table 5, those with history of TCA consumption had significantly higher parenteral morphine utilization (*p*<0.001). In the same way, the opium and methadone addiction was associated with increased parenteral morphine utilization (*p*<0.001). However, there was no association between morphine utilization and alcohol abuse, benzodiazepine consumption, allergy and smoking (Table 5). We have found that those with pain in the extremities probably due to trauma and those with chest pain had significantly higher parenteral morphine utilization according to the DDD (*p*<0.001). The utilization of the parenteral morphine was comparable between the other causes of admission (Table 6). The order of morphine prescription was intravenous in all patients except for 4 (1 in 2013, 2 in 2014, and 1 in 2015 years) that was intramuscular. The administered dosages by the physician were the same as the dose of the nursing staff in almost all the patients except in 3 patients (1 in 2013, 1 in 2014 and 1 in 2015 years), which the injected dosage was less than the that administered by the physician.

**Table 4:** The utilization of the parenteral morphine according to the DDD in different age and gender groups of 500 patients referred to the emergency department

**Gender**	**Morphine Utilization (DDD** ^c^ **)**			**p-value**
	**Lowest**	**Highest**	**Mean ± SD** ^d^	
Men	0.10	0.43	0.15 ± 0.06	>0.001^α^
Women	0.10	0.30	0.13 ± 0.04	
**Age **				
10-19	0.10	0.10	0.10 ± 0.03	0.061^β^
20-29	0.10	0.33	0.15 ± 0.06	
30-39	0.10	0.43	0.14 ± 0.06	
40-49	0.10	0.40	0.15 ± 0.06	
50-59	0.10	0.40	0.15 ± 0.06	
60-69	0.10	0.30	0.13 ± 0.05	
70-79	0.10	0.33	0.14 ± 0.07	
≥80	0.10	0.20	0.13 ± 0.04	

^α^Mann-Whitney test; ^β^Chi-square test;^ c^**DDD:** Defined Daily Doses; ^d^**SD:** Standard deviation

**Table 5:** The utilization of the parenteral morphine according to the DDD in different drug and substance abuse groups of 500 patients referred to the emergency department

**Variables**	**Morphine Utilization (DDD** ^c^ **)**			**p-value **
	**Lowest**	**Highest**	**Mean ± SD** ^d^	
**Alcohol**				
Yes	0.10	0.43	0.14 ± 0.06	0.128^α^
No	0.10	0.27	0.15 ± 0.05	
**TCA** ^e^				
Yes	0.10	0.43	0.14 ± 0.06	<0.001^α^
No	0.10	0.17	0.12 ± 0.03	
**Benzodiazepine**				
Yes	0.10	0.43	0.14 ± 0.06	0.087^α^
No	0.10	0.27	0.12 ± 0.05	
**Allergy**				
Yes	0.10	0.43	0.14 ± 0.06	0.075^α^
No	0.10	0.20	0.13 ± 0.04	
**Smoking**				
Yes	0.10	0.43	0.14 ± 0.05	0.085^α^
No	0.10	0.40	0.14 ± 0.06	
**Addiction**				
No	0.10	0.40	0.14 ± 0.05	<0.001^β^
Opium	0.10	0.40	0.20 ± 0.07	
Methadone	0.10	0.43	0.18 ± 0.08	
Others	0.10	0.17	0.12 ± 0.03	

^α^Mann-Whitney test; ^β^Chi-square test; ^c^**DDD:** Defined Daily Doses; ^d^**SD:** Standard deviation; ^e^**TCA:** Tricyclic anti-depressant

**Table 6:** The utilization of the parenteral morphine according to the DDD in different cause of admission of 500 patients referred to the emergency department

	**Morphine Utilization (DDD** ^b^ **)**			**p-value **
	**Lowest**	**Highest**	**Mean ± SD** ^c^	
Trauma (%)	0.10	0.33	0.13 ± 0.05	<0.001^α^
Dyspepsia (%)	0.10	0.33	0.14 ± 0.05	
Flank pain (%)	0.10	0.37	0.15 ± 0.06	
Headache (%)	0.10	0.17	0.11 ± 0.02	
Chest pain (%)	0.10	0.43	0.13 ± 0.10	
Back pain (%)	0.10	0.27	0.13 ± 0.05	
Limb pain (%)	0.10	0.40	0.17 ± 0.11	
Lethargy (%)	0.10	0.33	0.14 ± 0.07	
Testis pain (%)	0.10	0.10	0.10 ± 0.04	
Others (%)	0.10	0.17	0.11 ± 0.03	

^α^Chi-square test; ^b^**DDD:** Defined Daily Doses; ^c^**SD:** Standard deviation

## Discussion

Appropriate and reasonable prescribing of medicine is one of the important factors in providing health care for the community. Obviously, neglecting the rational use of the medication can lead to inappropriate consequences such as patient dissatisfaction, prolonged and exacerbated disease, dangerous side effects, hospitalization, reduced patient and doctor communication, and ultimately increased medical costs for individuals and government agencies, and most importantly causing a chronic shortage of drugs across the country [16,17]. Because of the side effects of excessive and incorrect use of the drug and its economic implications, today there is a strong tendency to curtail drug use internationally, especially in advanced countries. One of the main causes of overdose is the lack of information in both prescribing and consumer groups [18].

The current status of Iranian opiate drugs in terms of distribution and control, and in terms of clinical expenditure, does not enjoy the desired quality. In advanced countries, protocols for drug use are provided by competent authorities as standard and are available to hospitals and medical staff. In our country, the Ministry of Health and Medical Education has begun this process (though late) and has provided these protocols in the past few years, but we still see that the pattern of prescribing and taking narcotic drugs is deliberately illogical and outside of the established standards. Failure to consider these standards will cause many problems. Although the incidence and risks of narcotic drugs have so far been low, this phenomenon is limited to narcotic drugs, their very low dosages and the lack of accurate and organized reporting of side effects, drug dependence and medical-medical errors in our country. Is relevant [12].

The main points of the protocol used in this study, which was based on the World Health Organization guidelines based on DDD, morphine. The aim of this study was to determine the pattern of administration of morphine ampoule in the emergency department of Imam Khomeini Hospital in Sari from 2013 to 2016. Results from 500 patients referring to Imam Khomeini Hospital during the years 2013-2016, who received morphine, which shows the age range of 12 to 79 years with mean and standard deviation (48.29 ± 10.37). Of all patients, 306 (61.2%) were male and 194 (38.8%) were female. The prescription order of morphine was intravenous (IV) in all patients except for 4 cases (one case in year 92, two cases of 94 years and one in 95 cases) that was intramuscular (IM). Also, in all patients, the doses prescribed by the physician were the same as the dose of the nursing staff, and in only three cases (one case of 92 years, one in 1993 and one in 95 years), the nurse's injectable dose was less than the dose prescribed by the physician. Which is consistent with the method of prescribing the drug in the study of renown and colleagues [12], which is 97.5% of patients intravenously. But in Hajti *et al*., [11], anti-opioid analgesia was prescribed in prescriptions of physicians in most cases as needed (PRN) and intramuscularly, and in some cases it was prescribed other than PRN, and in most cases prescriptions prescribed by the physician There is a difference in the type of antinociception used by nurses. Intramuscular administration can relieve later pain in patients [19]. In more recent studies in other countries, intravenous and pacemaker (PCA) methods have been used to relieve pain in the patient better than the traditional method of intramuscular injection [20].

In the present study, there was a significant relationship between the average consumption of morphine based on DDD, gender, smoking and drug addiction. In most studies, gender has a significant relationship with injecting drug use [21, 22]. In Hajati *et al*., [23] Cigarette smoking is associated with an increase in overall drug use (DDD). This finding is also consistent with earlier studies that show that smokers and hospitalized patients who have been forced to stop smoking are more likely than non-smokers in the hospital to receive more opioid drugs. One of the reasons for this can be to avoid symptoms of nicotine smoking cessation syndrome such as anxiety and insomnia [24]. Of course, it should be noted that possible interference with the use of narcotic drugs, which may lead to increased liver metabolism of narcotic drugs. However, in the study of Shohrati and colleagues, no significant relationship was found between the sex of the patients and the average daily consumption of the narcotic drug; on the other hand, 200 of the patients studied had 2 patients (1%) with addiction and 17 (8.5%) patients Cigarettes, which again showed that the average amount of injectable drug was not significantly different from other patients. Of course, given that patients have been hospitalized in different parts of the hospital for a long time, this can be attributed to their access to narcotic drugs by their relatives, so the amounts obtained from the drug use in patients cannot be very reliable.

In the study of Hajati *et al*., [11] Age increasing with injecting drug use had a reverse effect on DDD / day sensation. In the study of fame and colleagues, this relationship was inversely and meaningfully. On the one hand, the response to narcotic drugs increases with age, and on the other hand, the duration of relieving pain increases with age. However, in the present study, there was no significant relationship between the different age groups and the amount of morphine consumption, which can be due to the type of patients referred, which in most cases was associated with trauma and abdominal and abdominal pain. Also, our study was based on the fact that patients admitted to the emergency department were examined, and eventually one day in a hospital emergency room, which could justify this difference. Unfortunately, a similar study that specifically addresses the use of morphine in a hospital emergency department has not been obtained. The only study that studies the pattern of injecting drug use in the entire hospital sector is Vatanpour *et al*., [15] at Amiralmomenin Hospital in Zabol. For DDD / 100 bed days, consumption is comparable in hospitals, indicating the amount of DDD used per 100 beds per day, which is in the study of Vatanpour *et al*., [15] For the Emergency Department at 36.87. Among all hospitals, it had the highest morphine consumption, which is similar to 37.22 in the present study, which shows that the morphine consumption in the emergency department of Imam Khomeini Hospital in Sari is based on the prescribed dose, DDD (30 milligrams).

We note some limitations to the study. In some cases, it was merely addiction, but the type of addiction was not recorded, so some cases were excluded from the study. Smoking rates were not cited except for a few cases. The morphine side effects were not seen in any part of the file. Some patients who had been prescribed morphine had left the study within a short time with personal satisfaction.

In conclusion, the utilization of parenteral morphine in the ED of our center was higher than the WHO standard dosage. The morphine utilization was associated with male gender, opium addiction and TCA consumption. Elaboration and implementation of standard protocols for pain control, training of the medical staff on how to manage and evaluate the drug, and adequate supervision from the distribution stage to the stage of prescribing to the patient, the use of non-oral and injectable drugs before taking narcotic drugs, non-narcotic analgesics and anesthetic drugs are necessary to reduce the utilization of parenteral morphine in ED. 

## References

[1] Ayalew MB, Tegegn HG, Abdela OA (2019). Drug Related Hospital Admissions; A Systematic Review of the Recent Literatures. Bull Emrg Trauma.

[2] Blackburn JL (1993). Impact of Drug Usage Review on Drug Utilisation. Pharmacoeconomics.

[3] Casamayor M, DiDonato K, Hennebert M, Brazzi L, Prosen G (2018). Administration of intravenous morphine for acute pain in the emergency department inflicts an economic burden in Europe. Drugs Context.

[4] Khosravani S, Handjani F, Alimohammadi R, Saki N (2017). Frequency of neurological disorders in bullous pemphigoid patients: a cross-sectional study. Int Sch Res Notices.

[5] Knapp DA (1991). Development of criteria for drug utilization review. Clin Pharmacol Ther.

[6] Hennessy S, Bilker WB, Zhou L, Weber AL, Brensinger C, Wang Y (2003). Retrospective drug utilization review, prescribing errors, and clinical outcomes. JAMA.

[7] Addis A, Rocchi F (2006). Drug evaluation: new requirements and perspectives. Recenti ProgMed.

[8] Organization WH (2002). Promoting rational use of medicines: core components.

[9] Olin B, Hebel S, Dombek C (2007). Drug Facts and Comparisons, 2007.

[10] Masters BR, John E. Bennett, Raphael Dolin, Martin J. Blaser (2016). Mandell, Douglas, and Bennett’s Principles and Practice of Infectious Diseases, (2015) Eds: John E.

[11] Hajati G MA, Salamzadeh J, Moshiri K (2006). A survey on the pattern of consumption of narcotic drugs in surgical wards of Ayatollah Taleghani Hospital. Journal of Dental School.

[12] Shohrati M, Hosseini M, Rahimian S, Mastanabadi H (2011). Unreasonable consumption of narcotic drugs following appendectomy and hernioraphy.

[13] Colman I, Rothney A, Wright SC, Zilkalns B, Rowe BH (2004). Use of narcotic analgesics in the emergency department treatment of migraine headache. Neurology.

[14] McCaffery M, Ferrell BR (1997). Nurses' knowledge of pain assessment and management: how much progress have we made?. J Pain SymptomManage.

[15] Vatanpour H, Sufi H, Jomeh NE, Khorramnia A, Bovand T, Vatanpour S (2016). Parenteral opioid analgesics utilization pattern in Amir-al-Momenin hospital, Zabol-Iran. International Journalof Medical Research & Health Sciences.

[16] WHO Colaborating Center for Drugs Statistics Methodology.

[17] Picón-Camacho SM, Marcos-Lopez M, Bron JE, Shinn AP (2012). An assessment of the use of drug and non-drug interventions in the treatment of Ichthyophthirius multifiliis Fouquet, 1876, a protozoan parasite of freshwater fish. Parasitology.

[18] Teymourzade E Bs, hoseini M, zare H, vafaei A (2013). Drug utilization in Iran. Hakim Health Systems Research Journal.

[19] Dinarvand R (2000). Situation of drug prescription in Tehran city. Hakim Research Journal.

[20] Latkin C, Friedman S (2012). Drug use research: drug users as subjects or agents of change. Subst Use Misuse.

[21] Abbasi Asl M, Salehi S, Zamani Esmailabadi S, Nikchi P, Soleimani F (2014). Fuzzy clustering of medical sciences universities in Iran on the basis of medical indices in 2008. Journal ofHealth Administration.

[22] Laing R (1990). Rational drug use: an unsolved problem. Tropical doctor.

[23] Einarson TR ( 1993). Drug-related hospital admissions. SAGE Publications Sage CA.

[24] Shalviri G, Mohammad K, Majdzadeh S, Gholami K (2005). Comparing epidemiological methods in detecting drug safety signal in IRAN. irje.

